# Simultaneous Detection of Inflammatory Biomarkers by SERS Nanotag-Based Lateral Flow Assay with Portable Cloud Raman Spectrometer

**DOI:** 10.3390/nano11061496

**Published:** 2021-06-05

**Authors:** Yang Li, Xiaojia Liu, Jiuchuan Guo, Yueting Zhang, Jinhong Guo, Xinggui Wu, Bo Wang, Xing Ma

**Affiliations:** 1 School of Materials Engineering, Shanghai University of Engineering Science, Shanghai 201620, China; liyang18124@163.com (Y.L.); 15039066601@163.com (Y.Z.); 2 Sauvage Laboratory for Smart Materials, School of Materials Science and Engineering, Harbin Institute of Technology (Shenzhen), Shenzhen 518055, China; 18b954089@stu.hit.edu.cn; 3 Shenzhen Bay Laboratory, No. 9 Duxue Road, Shenzhen 518055, China; 4 School of Communication and Information Engineering, University of Electronic Science and Technology of China, Chengdu 611731, China; uestc_gjc@163.com (J.G.); guojinhong@uestc.edu.cn (J.G.); 5 CloudMinds Inc., Shenzhen Bay Science and Technology Ecological Park, Shenzhen 100022, China

**Keywords:** lateral flow assay (LFA) strip, surface-enhanced Raman scattering (SERS), point-of-care testing (POCT), inflammatory biomarkers, core-shell nanoparticles, portable cloud Raman spectrometer

## Abstract

Inflammatory biomarkers are closely related to infectious diseases. However, traditional clinical tests of laboratory inspection are unable to achieve rapid and accurate detection of these biomarkers on-site due to shortcomings such as complex experimental operation, expensive equipment, and long test time. Herein, we proposed a lateral flow assay (LFA) strip based on surface-enhanced Raman scattering (SERS) nanotags (SERS-LFA strips) for the simultaneous and quantitative detection of dual infection biomarkers, serum amyloid A (SAA) and C-reactive protein (CRP), respectively. In practice, mesoporous silica (mSiO_2_)-coated Au nanoparticles (Au NPs) were used as the SERS substrate. Mercaptobenzoic acid (MBA) was embedded in the internal gap between Au NPs and the mSiO_2_ shell to prepare Au^MBA^@mSiO_2_ NPs, onto which SAA and CRP antibodies were modified to prepare two Au^MBA^@mSiO_2_ SERS nanotags. The Raman intensities of the test and control lines were simultaneously identified for the qualitative detection of SAA and CRP, with limits of detection (LODs) as low as 0.1 and 0.05 ng/mL for SAA and CRP, respectively. Finally, aiming at point-of-care testing (POCT) applications, we used a smartphone-based portable Raman spectrometer to quantitatively analyze the SERS-LFA strips. The Raman signal could still be accurately detected when the concentration of SAA and CRP was 10 ng/mL, which is lower than the LOD required in clinical practice for most diseases. Therefore, taking into account its simple operation and short analysis time, by using a portable Raman spectrometer which can be equipped with a 5G cloud-based healthcare management system, the current strategy based on SERS-LFA provides the potential for the quick and on-site diagnosis of infectious diseases such as sepsis, which is of great significance for medical guidance on the treatment of widely spread infection-related diseases in remote areas that lack well-developed medical resources.

## 1. Introduction

A series of inflammatory biomarkers such as CRP, SAA, procalcitonin (PCT) and interleukin 6 (IL-6) have been found to be associated with infectious diseases and have been used to diagnose sepsis [1,2,3,4,5,6]. Recently, the first example of a SERS-based assay for the detection of fibroblast activation protein (FAP), an important marker of inflammation in the gastrointestinal tract during the fibrotic evolution of Crohn’s disease, and also a marker of CAFs (cancer-associated fibroblasts), was proposed [7]. Recent reports indicate that the death rate caused by sepsis is as high as 50%, affecting more than 30 million people worldwide each year [8]. Sepsis is a life-threatening organ dysfunction caused by the body’s inflammatory response to infections such as bacteria, fungi and viruses. Blood culture, blood count and polymerase chain reaction are all routine diagnostic methods [8,9,10,11]. Pathogenic bacteria and viruses can cause people to suffer from common infectious diseases, and there are significant differences between the two in clinical medication [12,13]. Due to the lack of specific symptoms, one clinical difficulty is to distinguish between bacterial and viral infections. In the case of viral infection, the concentration of SAA in human blood increases significantly, while the concentration of CRP does not change significantly. Within a few hours after bacterial infection, the concentration of SAA and CRP in human blood increases significantly and induces an inflammatory response in the human body [14]. However, the increase in the concentration of SAA is higher than that of CRP, indicating that SAA is more sensitive than CRP. After treatment with medication, among many patients, the SAA concentration in the blood of those with viral infections decreased faster than those with bacterial infections [6]. Therefore, the simultaneous and quantitative detection of SAA and CPR can identify bacterial and viral infections and offer proper guidance on clinical medication [15].

Traditional diagnostic methods for infectious diseases include erythrocyte sedimentation rate, pathogen culture, white blood cell count and PCR [3,16,17,18]. However, these methods for treating infectious diseases such as sepsis are unable to achieve fast and accurate diagnosis due to shortcomings including complex experimental operation, expensive equipment, long test time and the need for professional operators. Therefore, point-of-care testing (POCT) technology has become one of the most important technologies for health monitoring because it can quickly, accurately and conveniently obtain health information. The lateral flow assay (LFA) based on nanotags has become one of the most widely used POCT technologies due to its simple operation and rapid detection [19,20,21,22,23]. Currently, a number of nanomaterials are used as nanotags, such as quantum dots (QD), dye-loaded NPs, SERS nanomaterials, magnetic NPs, up-converting NPs (UCNPs) and so forth [8,20,24,25,26], among which SERS nanotags show great potential as a signal label for LFA. For instance, Zhang et al. proposed an LFA based on SERS nanotags for the multiplex and quantitative detection of three cardiac biomarkers, Myo, cTnI and CK-MB, for the early diagnosis of acute myocardial infarction [19]. Wang et al. developed a sensitive SERS-based strip for the detection of influenza A H1N1 virus and HAdV by using Fe_3_O_4_@Ag SERS nanotags [25]. Liu et al. reported a novel SERS-based LFA strip for the ultrasensitive analysis of SAA and CRP by using Fe_3_O_4_@Au SERS nanotags [6]. Among these works, core-shell SERS nanotags have become a promising type of nanotag because of their extremely high sensitivity, excellent stability and the specificity of the SERS signal.

**Figure 1:**
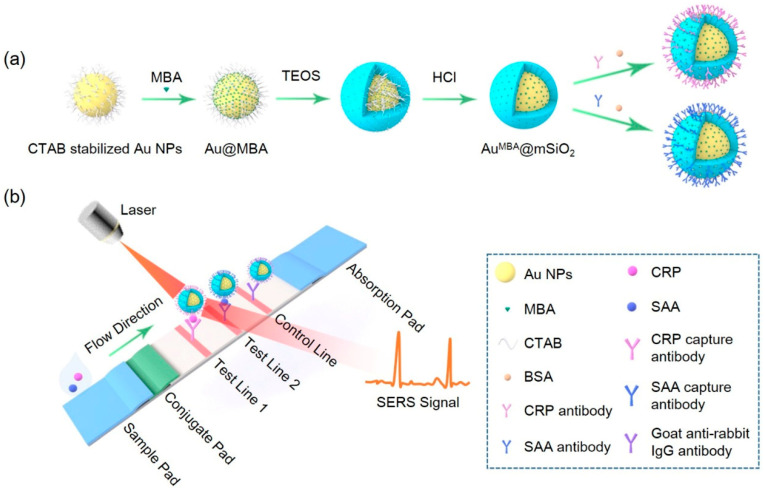
(**a**) The preparation flow chart of the core-shell Au^MBA^@mSiO_2_ nanotags; (**b**) the principle of the SERS-LFA strip for detection of SAA and CRP.

In this work, we propose an LFA strip based on core-shell Au^MBA^@mSiO_2_ SERS nanotags for the simultaneous and quantitative detection of SAA and CRP, respectively (Figure 1). SAA and CRP antibodies are modified on Au^MBA^@mSiO_2_ NPs, and two core-shell Au^MBA^@mSiO_2_ SERS nanotags with excellent chemical stability and SERS activity are prepared. The entire assay process is completed in 20 min, which demonstrates its outstanding sensitivity and accuracy for the quantification of SAA and CRP at the same time. Compared with other similar approaches, the nanoparticles and nanotags prepared by us are stable in terms of their physical and chemical properties, easy to synthesize and have good dispersion in solution. The SERS-LFA strips prepared by us have high sensitivity and specificity to SAA and CRP antigens, and the LOD of the SERS-LFA strips is low, which means that they can realize the detection of lower-concentration antigens. Especially in the current context of the novel coronavirus, the method of rapid antigen detection based on strips is particularly important, especially in remote areas where medical conditions are insufficient. Based on our proposed method, using test strips to quickly identify and quantify antigens is fast and efficient. More importantly, we further use a portable Raman spectrometer to quantitatively analyze the SERS-LFA strip, which can give reliable results for clinical diagnosis. Taking account of the online healthcare management system coupled with the smartphone-based Raman spectrometer, the current work demonstrates significant potential for the clinical diagnosis and corresponding online medical guidance of infectious diseases in remote areas.

## 2. Materials and Methods

### 2.1. Materials

Hexadecyltrimethylammonium bromide (CTAB, 99%), sodium hydroxide (NaOH), formaldehyde solution (37 wt.%), HAuCl_4_, Mercaptobenzoic acid (MBA, 90%), tetraethyl orthosilicate (TEOS), 3-aminopropyl triethoxysilane (APTES, 99%), *N*,*N*-dimethylformamide (DMF), bovine serum albumin (BSA, 96%), succinic anhydride (99%), triethylamine (99%), HCl (37 wt.%), ethyl dimethylaminopropyl carbodiimide hydrochloride (EDC, 98%), *N*-hydroxysuccinimide (NHS, 97%), anhydrous ethanol and PBS (10×) were purchased from Sigma-Aldrich (St. Louis, MO, USA). The types of nitrocellulose (NC) membrane film were CN 140 and Pall Vivid 90 (Shanghai Aiyan Biotechnology Co., Ltd. (Shanghai, China)). Absorbent paper was not treated and glass fiber was treated with running buffer in advance. Deionized (DI) water was prepared with a Millipore Milli-Q system (Sinoinstrument Co., Ltd. (Guangzhou, China)). Rabbit IgG and goat anti-rabbit IgG antibodies were purchased from Hangzhou Clongene Biotech Co, Ltd. (Hangzhou, China). Monoclonal anti-CRP antibody (Cat #4C28-C6), monoclonal anti-CRP antibody (Cat #4C28-C2), monoclonal anti-SAA antibody (K8d6) and monoclonal anti-CRP antibody (K7c5) were purchased from Okay Biotechnology Co., Ltd. (Nanjing, China).

### 2.2. Instruments

Transmission electron microscopy (TEM) images of SERS nanotags were captured by TEM (FEI Tecnai G2, FEI, Eindhoven, The Netherlands). UV–vis adsorption spectra were collected by a spectrometer (UV-2600, Shimadzu Corporation, Shimadzu, Japan). The diameter and zeta potential values of nanoparticles were measured on a ZetaSizer (Malvern, UK). The corresponding brightness values of the test and control lines of the SERS-LFA strips were analyzed by ImageJ software. Raman spectra of SERS nanotags and test lines were obtained on a confocal Raman spectrometer (Horiba Instruments (Shanghai) Co., Ltd. (Shanghai, China)) and a portable smartphone-based Raman spectrometer (Cloudoptek Technology Co., Ltd. (Beijing, China)). The portable Raman spectrometer is based on a handheld smartphone, which is small in size and easy to carry. The upper left of the device is equipped with a laser, the maximum power of the laser is 500 mW, and the laser wavelength is 780 nm. The device can detect solids, liquids, etc. After capturing the Raman spectrum data, they will be compared with the pre-imported spectrum database, so that they can distinguish substances to a certain extent. The device has a built-in Bluetooth module, which can remotely transmit the obtained Raman information to other devices through the 5G network.

### 2.3. Synthesis of Core-Shell Au^MBA^@mSiO_2_ Nanoparticles

First, 0.15 g CTAB and NaOH solution (50 mM, 1.8 mL) were added to 72 mL DI water and the mixture was heated to 80 °C with stirring using a magnetic stirrer. The formaldehyde solution (3.0 mL, 3.7 wt.%) was added to the above solution. After stirring for 10 min, an aqueous HAuCl_4_ solution (2.4 mL, 50 mM) was added. MBA alcohol solution (10 μM, 3 mL) was added to the solution and stirred for 10 min. Then, a certain amount of TEOS was added. After 1 h of reaction, the product was centrifuged, washed with DI water 3 times and further washed with anhydrous ethanol once. Finally, an appropriate amount of HCl (37 wt.%) was added to the ethanol suspension of the as-prepared Au^MBA^@mSiO_2_ NPs to remove the surfactant template of CTAB [27].

### 2.4. Preparation of Core-Shell Au^MBA^@mSiO_2_ SERS Nanotags

First, 10 mg Au^MBA^@mSiO_2_ NPs was uniformly dispersed in 10 mL ethanol. Next, 100 μL APTES was added and stirred at room temperature for 24 h to yield Au^MBA^@mSiO_2_-NH_2_. After centrifugation, the product was washed 3 times with DMF and evenly dispersed in 10 mL DMF. Then, 50 mg of succinic anhydride and 50 μL of triethylamine were added to the solution and stirred at room temperature for 24 h. Thus, the carboxyl group was modified on the surface of Au^MBA^@mSiO_2_ NPs. Afterwards, 1 mg of Au^MBA^@mSiO_2_-COOH NPs was activated with EDC and NHS, and 0.25 mg of three antibodies (anti-SAA, anti-CRP and rabbit IgG antibodies) was added to the above solution and stirred for 4 h. Finally, 100 μL of BSA solution (10 wt.%) was then added and stirred for 1 h.

### 2.5. Preparation of SERS-LFA Strip

The SERS-LFA strip consisted of a sample pad, a conjugate pad, a NC membrane, an absorption pad and a plastic backing pad. Sample pad and conjugate pad were made of glass fiber, soaked in a buffer solution and then dried at 37 °C overnight. As the NC membrane was at the bottom of the strip, the NC membrane was pasted onto the plastic backing pad and closed to the conjugate pad. The other end of the NC membrane was pasted with an absorption pad that had not undergone any pretreatment, and the end of the absorption pad kept an overlap of around 4 mm with the NC membrane and was tightly attached to the NC membrane. The solutions containing three Au^MBA^@mSiO_2_ NPs labeled with antibodies were uniformly mixed at a volume ratio of 1:1:1. Then, the XYZ Platform Dispenser (Model: HM3035, Shanghai Kinbio Tech. Co., Ltd. (Shanghai, China)) was used to spray the solutions onto the conjugate pad at a speed of 3 μL/cm. The XYZ Platform Dispenser was driven by the motor on the X axis (vertical position), Y axis (horizontal position) and Z axis (the position of the sliding tip and the height of the platform), and 3D positioning was performed through the digital input operation interface to spray the solutions on the conjugate pad and NC membrane at a uniform speed according to the preset spraying speed. SAA capture (1 mg/mL), CRP capture (1 mg/mL) and goat anti-rabbit IgG (1 mg/mL) antibodies were sprayed onto the NC membrane at a speed of 1.5 μL/cm to generate two test lines and one control line, with an interval distance of around 6 mm. After the conjugate pad and the NC membrane were processed, they were dried at 37 °C overnight. The ends of each component overlapped, ensuring a continuous flow by capillary attraction of the developing solution from the sample pad to the absorption pad [19]. The strips were cut into sections of 4 mm in width by a CNC cutting machine and stored in an airtight bag containing desiccant for further use.

### 2.6. Optimization of the SERS-LFA Strip

Before the preparation of the SERS-LFA strips, parameters such as the pH value, the types of glass fiber and running buffer, the volume of the analytes dropped on the sample pad, concentration and speed during spraying of nanotag solution and capture antibodies solution were all optimized. The presence of surfactant in the running buffer and the pore size of the NC membrane are both important factors regulating the binding time between antigen and antibody and affecting the sensitivity of the LFA [28]. The glass fiber types used in the SERS-LFA strips were SB08 and RB65. The running buffer types of processed fiberglass included YX1 (12.1 g/L Tris, 10 g/L PVP, 10 g/L S9, 5 g/L Casein, 0.2 g/L NaN_3_, pH = 8.0), YX2 (19.05 g/L borax, 10 g/L PVP, 6 g/L Tris, 10 g/L S9, 5 g/L sodium cholate, 1 g/L Casein, 0.2 g/L NaN_3_, pH = 8.5), YX3 (19.05 g/L borax, 10 g/L PVP, 10 g/L S9, 3 g/L EDTA, 1 g/L Casein, 0.2 g/L NaN_3_, pH = 9.0). There were 12 combinations of 2 kinds of glass fiber, 2 kinds of NC membrane and 3 kinds of running buffer, which could be used to prepare 12 different SERS-LFA strips. After the sample pads were dropped with analytes for the immune experiment, the SERS-LFA strip with the deepest and most uniform color and the widest width of the test and control lines was selected for the SERS-LFA strip. After many immune experiments, we used RB65 glass fiber treated with YX1 running buffer and CN140 NC membrane to prepare the SERS-LFA strips. We confirmed that the optimal spraying concentrations of nanotag solution and capture antibodies solution were 3 and 1 mg/mL, respectively. The spraying speeds of the nanotag solution and capture antibodies solution were identified to be 3 and 1.8 μL/cm, respectively. The volume of the analytes dropped onto the sample pad was fixed to 90 μL.

### 2.7. Procedures of Simultaneous and Quantitative Detection of SAA and CRP by SERS-LFA Strip

As shown in Figure 1b, 90 μL of the analyte solution was dropped onto the sample pad. Under the capillary attraction of the SERS-LFA strip, the analyte solution migrated along the sample pad toward the absorption pad. When the analytes containing SAA and CRP flowed through the conjugate pad, due to the antibody–antigen reaction, the SAA and CRP quickly bound with the corresponding detection antibodies modified on the nanotags pre-sprayed on the conjugate pad, forming Au^MBA^@mSiO_2_-SAA and Au^MBA^@mSiO_2_-CRP immune complexes. These two immune complexes continued to migrate with the solution, and when they reached two test lines, the Au^MBA^@mSiO_2_-CRP immune complex was captured by the CRP capture antibody pre-immobilized on test line 1 and the Au^MBA^@mSiO_2_-SAA immune complex was captured by the SAA capture antibody pre-immobilized on the test line 2. As a result, the two immune complexes with a sandwich structure were formed on the two test lines. Finally, Au^MBA^@mSiO_2_ NPs modified with rabbit IgG antibody continued to migrate. When they reached the control line, they were captured by the goat anti-rabbit IgG antibody pre-immobilized on the control line. With the nanoparticles accumulating, the test line and control line showed a characteristic red band. Regardless of the concentration of SAA and CRP in the analyte solution, the control line of each SERS-LFA strip was visible to the naked eye and had almost the same color, which could be used to verify whether the SERS-LFA strip was working properly. Because the number of nanoparticles accumulated on the control line was only related to the amount of rabbit IgG antibody pre-immobilized on the conjugate pad, the presence of the two biomarkers could be confirmed both through color change and the Raman signal intensity of the corresponding test lines [19]. The qualitative detection of the two biomarkers was judged by the presence or absence of the red band of the corresponding test lines, and the quantitative detection was achieved by measuring the Raman signal intensities and brightness values of the corresponding test lines.

## 3. Results and Discussion

### 3.1. Fabrication and Characterization of Core-Shell Au^MBA^@mSiO_2_ SERS Nanotags

**Figure 2:**
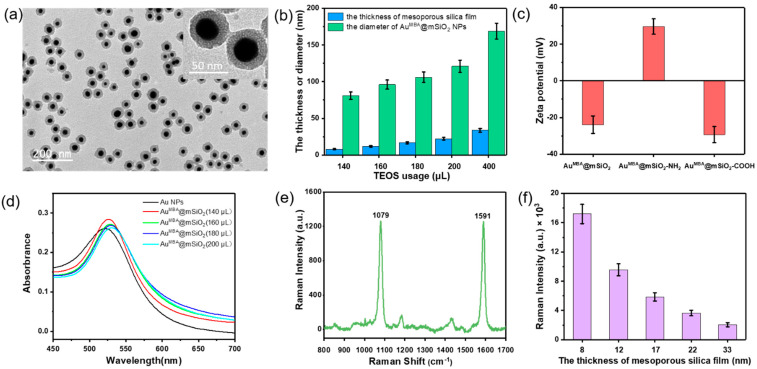
Characterization results of the Au^MBA^@mSiO_2_ NPs and corresponding SERS nanotags. (**a**) TEM images of Au^MBA^@mSiO_2_ NPs (TEOS usage: 140 μL). (**b**) During the preparation of Au^MBA^@mSiO_2_ NPs, the relationship between TEOS usage (140, 160, 180 and 200 μL) and the thickness of mesoporous silica film and the diameter of Au^MBA^@mSiO_2_ NPs (measured by a ZetaSizer) was evaluated. Error bars represent the standard deviation (N = 50). (**c**) During the preparation of SERS nanotags, the zeta potentials of Au^MBA^@mSiO_2_, Au^MBA^@mSiO_2_ -NH_2_, Au^MBA^@mSiO_2_ NPs (N = 10) were measured. (**d**) UV-vis absorption spectra of Au NPs and Au^MBA^@mSiO_2_ nanoparticles with different TEOS usage. (**e**) Raman spectra of Au^MBA^@mSiO_2_ NPs (TEOS usage:140 μL) characterized by a confocal Raman spectrometer. (**f**) The relationship between total Raman intensities at 1079 cm^−1^ of ten Au^MBA^@mSiO_2_ NPs and the thickness of mesoporous silica film.

As shown in Figure 1a, the process of the preparation of the SERS nanotags mainly consists of five steps, as detailed in the Materials and Methods section. The Au NPs were prepared by reducing HAuCl_4_ with formaldehyde according to a previous report with proper modifications [27]. MBA molecules were modified on the surfaces of Au NPs by forming Au-S bonds. Then, TEOS was hydrolyzed under alkaline conditions to form a mesoporous silica layer on the surfaces of Au NPs. The TEM images of Au^MBA^@mSiO_2_ NPs are shown in Figure 2a and Figure S1a–e. The thickness of the mesoporous silica film gradually increased with the increase in TEOS usage, resulting in a significant increase in the diameter of the nanoparticles (Figure 2b). When the amount of TEOS was less than 140 μL, the amount of TEOS hydrolyzed was lower, making it difficult for mSiO_2_ to coat the nanoparticles. Even if a small amount of mSiO_2_ was coated on the nanoparticles, the mSiO_2_ layer was very uneven. Therefore, the amount of TEOS was at least 140 μL.

During the preparation process, the size of the Au NPs can be adjusted by changing the amount of formaldehyde. The thickness of the mSiO_2_ layer is also adjustable by changing TEOS usage [27]. UV-vis absorption spectra of Au NPs and Au^MBA^@mSiO_2_ NPs with different TEOS usage are shown in Figure 2d. The obtained Au NPs displayed an absorption band at 524 nm. For Au^MBA^@mSiO_2_ NPs with mesoporous silica films of different thicknesses, a certain degree of red shift of the absorption band from 524 nm to 535 nm was observed. The gradual increase in the thickness of the mSiO_2_ film resulted in a change in the local refractive index, resulting in a slight red shift [29]. Au NPs easily agglomerated during the process of preparation and centrifugation, and the existence of the passivate mSiO_2_ film could solve this problem well, so that the nanoparticles had better dispersibility in the solution. The existence of the mSiO_2_ film separated the MBA molecule from other parts, avoiding the influence of other molecules on the MBA molecule, and it could be used as a chemically active film for further functionalization of the amino and carboxyl groups, and for further modifying the antibody to make the SERS nanotags. Figure 2c shows the zeta potentials of Au^MBA^@mSiO_2_, Au^MBA^@mSiO_2_-NH_2_, Au^MBA^@mSiO_2_-COOH NPs during the preparation of the nanotags. The corresponding changes in the surface potential of the nanoparticles indicated the successful modification of the amino and carboxyl groups on the Au^MBA^@mSiO_2_ NPs. EDC and NHS coupling reactions were conducted to conjugate the carboxyl group on the surface of Au^MBA^@mSiO_2_ NPs with SAA and CRP antibodies [19].

### 3.2. Raman Signal of Au^MBA^@mSiO_2_ SERS Nanotags

Figure 2e shows the SERS spectrum of MBA embedded in Au^MBA^@mSiO_2_ SERS nanotags. As a SERS substrate material, Au NPs could interact with MBA and could significantly amplify the Raman signal of MBA to achieve a good SERS effect. MBA has two obvious SERS peaks at 1079 and 1591 cm^−1^, respectively, which represent ring deformation vibration coupled with C-S stretching and carboxyl asymmetric stretching vibration coupled with C-C stretching. We chose the SERS characteristic peak at 1079 cm^−1^ to quantify the intensity of the Au^MBA^@mSiO_2_ SERS nanotags. As shown in Figure 2f, we measured the average Raman intensities at 1079 cm^−1^ of Au^MBA^@mSiO_2_ NPs prepared with different levels of TEOS usage. With the usage of TEOS decreasing, the thickness of the mSiO_2_ film gradually decreased, and the SERS intensities of Au^MBA^@mSiO_2_ NPs increased significantly. The reason could be associated with the “shielding” effect of the mSiO_2_ film, which interferes with the exciting laser and scatters the SERS signal light. The reason is that hot spots were formed between the Au core and the mesoporous silica film when the mesoporous silica film was very thin. Due to the thin film, most of the laser could pass through the mesoporous silica film to the surfaces of the Au nanoparticles, interact with MBA molecules and cause the particles’ Raman signal to increase sharply. However, when the mesoporous silica film was thicker, the optical path in the mesoporous silica shell was increased, and the laser scattering effect was enhanced, so that the laser intensity was consumed to a certain extent, so that the SERS intensity of the nanoparticles was significantly reduced [29]. We measured the SERS intensities at 1079 cm^−1^ of five different batches of Au^MBA^@mSiO_2_ SERS nanotags (TEOS usage: 140 μL), and 10 different nanoparticles of each batch were selected. The relative standard deviation (RSD) of the SERS intensities of all nanoparticles was 4.6%, indicating that the SERS nanotags had excellent reproducibility. As shown in Figure S2c, the Raman intensity of a single Au^MBA^@mSiO_2_ NP was stronger than that of a single Au^MBA^@mSiO_2_ nanotag due to the modification of chemical groups and antibodies on the surface of the nanoparticle. With the extension of the storage time of Au^MBA^@mSiO_2_ NPs and nanotags, their Raman intensities decreased to a certain extent. After 50 days of storage, their Raman intensities still exceeded 70% of the Raman intensities of newly prepared Au^MBA^@mSiO_2_ nanoparticles, which indicates the good stability of the SERS nanotags.

### 3.3. Sensitivity and Specificity of the SERS-LFA Strip

**Figure 3:**
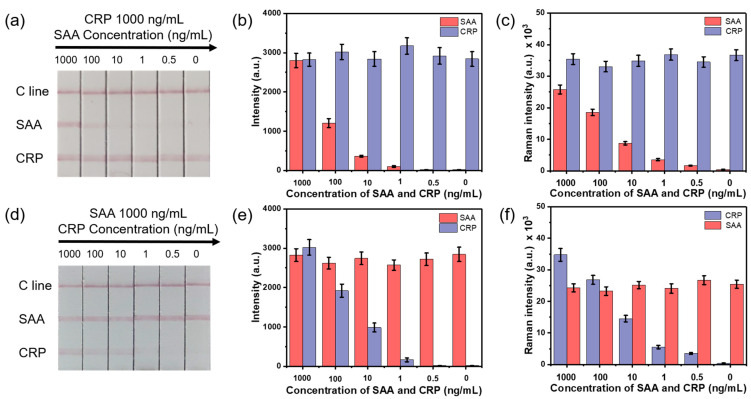
Sensitivity and specificity of the Au^MBA^@mSiO_2_-based LFA strips. (**a**,**d**) Photographs of the test LFA strips. (**b**,**e**) The corresponding brightness values of two test lines and (**c**,**f**) the corresponding Raman intensities at 1079 cm^−1^ of two test lines. The error bars indicate the standard deviations calculated from five separate experiments.

The sensitivity of the Au^MBA^@mSiO_2_-based SERS-LFA strip was evaluated by detecting PBS solution containing different concentrations of CRP and SAA. First, 90 μL of CRP and SAA solution of different concentrations were added onto the sample pad. The detection results were observed by the naked eye and also quantitatively analyzed by a confocal Raman spectrometer. As the concentration of SAA and CRP dropped on the sample pad gradually decreased, the color of the two test lines gradually became lighter until they were invisible to the naked eye. The visual limit of detection (vLOD) for qualitative evaluation was defined as the concentration of the SAA and CRP corresponding to the lightest color of the test line visible to the naked eye. The vLODs of the two test lines of SAA and CRP were 10 and 1 ng/mL, respectively (Figure 3). This indicated that the binding ability of CRP antibody and antigen was stronger than SAA, so CRP had higher detection sensitivity [30]. Therefore, the current system can first of all directly serve as a conventional LFA strip for the qualitative identification of target analytes through color change.

In order to detect SAA and CRP inflammation biomarkers simultaneously and quantitatively, the specificity of the proposed SERS-LFA strip should be tested. The specificity, expressed as cross-reactivity, was evaluated by monitoring the change in the Raman intensities of the test lines when changing the concentration of SAA and CRP dropped on the sample pad. Two sets of samples were prepared. One set was to mix CRP with a constant concentration of 1000 ng/mL while varying the concentration of SAA (1000, 100, 10, 1, 0.5 ng/mL). The other was to mix SAA with a constant concentration of 1000 ng/mL while varying the concentration of CRP (1000, 100, 10, 1, 0.5 ng/mL) [19]. The confocal Raman spectrometer was equipped with a 50× objective lens, and the laser wavelength of the equipped laser was 633 nanometers. The Raman strength of the test line was obtained by scanning a rectangular area of the test line. The scanning area was an area of 80 μm × 80 μm, which contained 20 × 20 pixels, and the total time for signal integration was 2400 s. The test results of the LFA strips are shown in Figure 3. Figure 3a,d show photographs of the SERS-LFA strips after the test. The corresponding Raman intensities of the SERS-LFA strips in Figure 3a,d are shown in Figure 3c,f, respectively. The corresponding brightness values of the test lines of the SERS-LFA strips in Figure 3a,d analyzed by ImageJ software are shown in Figure 3b,e, respectively. The brightness curves of two test lines and one control line of the SERS-LFA strips are shown in Figure S3. Regardless of existing of SAA/CRP, the control lines of all strips were all red, indicating that the results were all valid. As shown in Figure 3a, when keeping the CRP concentration constant and with the SAA concentration gradually decreasing, the test line 1 (CRP) of each SERS-LFA strip appeared almost the same, with a red color. The comparison of five experiments showed that there was no obvious change in the corresponding Raman intensities at 1079 cm^−1^ (Figure 3c) and the brightness values (Figure 3b) on the test line 1. The color of the test line 2 (SAA) gradually darkened, and the corresponding Raman intensities at 1079 cm^−1^ and brightness values gradually decreased with the SAA concentration gradually decreasing. Similarly, as the SAA concentration was constant and the CRP concentration gradually decreased (Figure 3d), the test line 2 of each SERS-LFA strip appeared almost the same, with a red color. The corresponding Raman intensities at 1079 cm^−1^ and the brightness values were equivalent (Figure 3e,f). The color of test line 1 gradually darkened, and the corresponding Raman intensities at 1079 cm^−1^ and brightness values gradually decreased with the CRP concentration gradually decreasing. The comparison of the five experiments showed that the relative standard deviation (RSD) values of the Raman intensities of the test line 1 and 2 were 9.8% and 13.2%, respectively, and the RSD values of the brightness values of the test line 1 and 2 were 10.9% and 14.8%, respectively. Typically, when using a Raman microscope equipped with a 50× objective, the size of the laser spot is a few square micrometers. This size is close to the size of the pores in the membrane. Due to the porosity of the membrane and the uneven distribution of particles, the RSD of the SERS intensity is usually close to 10–20%, and sometimes even more than 20%, especially in the case of low antigen concentrations. Therefore, it is necessary to perform Raman mapping on a larger section of the test area and perform multiple measurements to determine and reduce the RSD of the Raman signal. In the end, the RSDs of the SERS intensity of test lines 1 and 2 were 7.1% and 8.6%, respectively, which proved that the proposed SERS-LFA strips had excellent stability and specificity [6]. Therefore, the negligible cross-reactivity of the two biomarkers made it possible to use the proposed SERS-LFA strips to detect CRP and SAA simultaneously and quantitatively. It is worth noting that when the concentration of SAA and CRP was lower than 1 ng/mL, the colors of the two test lines were invisible to the naked eye, with brightness values of almost 0, but their Raman intensities were still identified with notable values. Therefore, compared with brightness, the Raman signal used for the quantitative detection of SAA and CRP had higher detection sensitivity, which could make the experimental results more accurate and shows the advantages of high sensitivity of the SERS nanotags.

### 3.4. Simultaneous and Quantitative Detection of CRP and SAA Based on the SERS-LFA Strip

**Figure 4:**
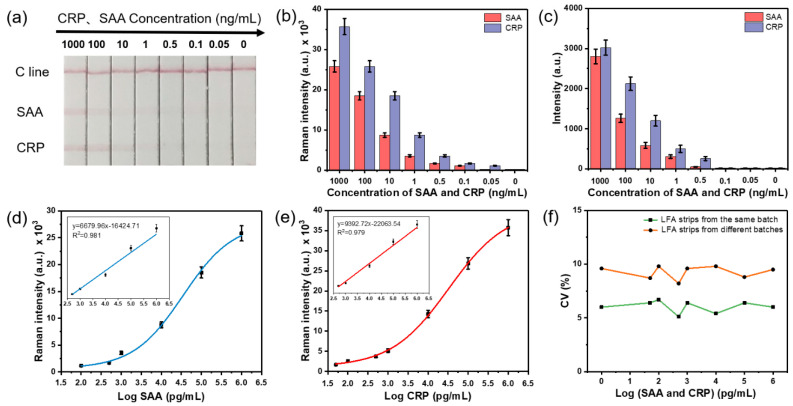
(**a**) Photograph of the test SERS-LFA strips at different SAA and CRP concentrations. (**b**) The corresponding Raman intensities at 1079 cm^−1^ and (**c**) brightness values (by ImageJ) of two test lines. The Raman intensities and calibration curves of the corresponding test lines of (**d**) SAA and (**e**) CRP. The insets in (**d**,**e**) show the linear relationship of the calibration curve within a certain concentration range. (**f**) Coefficients of variation (CVs) of the SERS-LFA strips for simultaneous and quantitative detection of different concentrations of SAA and CRP. The error bars indicate the standard deviations calculated from five separate experiments.

Through the above study, we found that the SERS-LFA strips had good detection sensitivity and specificity for SAA and CRP in a certain concentration range (0.5–1000 ng/mL). Next, we used the proposed SERS-LFA strips to simultaneously and quantitatively detect SAA and CRP with a wider concentration range (0.05, 0.1, 0.5, 1, 10, 100, 1000 ng/mL). As shown in Figure 4a, as the concentration of SAA and CRP dropped on the sample pad gradually decreased, the color of the two test lines gradually darkened until invisible to the naked eye. Shown in Figure S2a,b are the SEM images of test strips after immunoassays with SAA and CRP concentrations of 1000 and 0 ng/mL, respectively. When the SAA and CRP concentration was 1000 ng/mL for the immune experiment, there was a certain number of immune complexes in the test line area of the strip. The immune complexes may be larger clusters formed by the agglomeration of multiple nanoparticles, because these clusters appeared to be much larger than individual nanoparticles. In Figure S2b, only the fibrous structure of the NC membrane and the small holes between the fibrous structure can be seen. The vLODs of SAA and CRP were 10 and 1 ng/mL, respectively, which was consistent with the previous study on the specificity of the SERS-LFA strips. The brightness values analyzed by ImageJ software and Raman intensities measured by a confocal Raman spectrometer of the test lines were considered to realize the simultaneous and quantitative detection of SAA and CRP.

As shown in Figure 4b, the Raman intensities of the test lines gradually decreased with the concentration of SAA and CRP decreasing. Under the test conditions described in the article, we define the LOD of the test strip as the lowest concentration of SAA and CRP corresponding to the total Raman intensity value of the test strip exceeding 1000. When the concentration of SAA and CRP was 0.05 ng/mL, the Raman intensity of SAA was numerically lower than 1000, while the Raman intensity of CRP was numerically higher than 1000, which indicated that the LODs of SAA and CRP by the Raman signal were 0.1 ng/mL and 0.05 ng/mL, respectively. As shown in Figure 4c, the brightness values of the test lines gradually decreased with the concentration of SAA and CRP decreasing. Additionally, the LODs of SAA and CRP by brightness value were both about 0.5 ng/mL. The LODs of the quantitative detection of SAA and CRP using the Raman signal were arounds 5–10 times those of the brightness value. Therefore, the SERS-LFA strip had a wider detection range, lower LOD and higher detection sensitivity when using the SERS nanotag-based Raman signal. Based on the measurement of the Raman intensities of the test lines, the calibration curves for SAA and CRP were obtained and are shown in Figure 4d,e, respectively. The insets in Figure 4d,e show the linear curves of SAA (*R*^2^ = 0.981) and CRP (*R*^2^ = 0.979) and the corresponding linear regression equations, respectively. We can see that SAA and CRP have a good linear relationship between the concentration of SAA and CRP and the Raman intensity when the concentration range is 0.5–1000 ng/mL. The LODs of SAA and CRP were 0.1 and 0.05 ng/mL, respectively, which are consistent with the previous research content in this paper. It is worth noting that the LODs of the proposed SERS-LFA strip for the simultaneous and quantitative detection of SAA and CRP are lower than the LOD required by the clinic.

The coefficient of variation (CV) is a key factor that reflects the degree of dispersion of a set of data, and it is numerically equal to the ratio of the standard deviation to the average value of the data. In order to evaluate the bias among the prosed SERS-LFA strips of the same batch and different batches, the CVs of the SERS-LFA strips for each concentration of SAA and CRP were calculated. As shown in Figure 4f, the CVs of the SERS-LFA strips from same batch are all less than 8% for different concentrations of SAA and CRP, and the CVs of the SERS-LFA strips from different batches are all less than 10% for different concentrations of SAA and CRP, indicating that the SERS-LFA strips work well and the data are reliable.

After dropping the analytes on the sample pad of the SERS-LFA strip, the color change on the test lines could be observed in around 10 min, and the detection of two inflammatory biomarkers could be achieved within 20 min. We summarized the main analytical performance of our SERS-LFA strip (Table 1), and, compared with other reports, it is obvious that our SERS-LFA strips are comparable to or even better than most strips in terms of LOD and detection time. Moreover, by preparing SERS nanotags modified with other different antibodies, our SERS-LFA strip can also readily be used to detect many other biomarkers, such as PCT, IL-6 and other inflammation biomarkers, cardiac biomarkers, tumor biomarkers and so on.

### 3.5. Simultaneous and Quantitative Detection of CRP and SAA by Portable Cloud Raman Spectrometer for POCT

**Figure 5:**
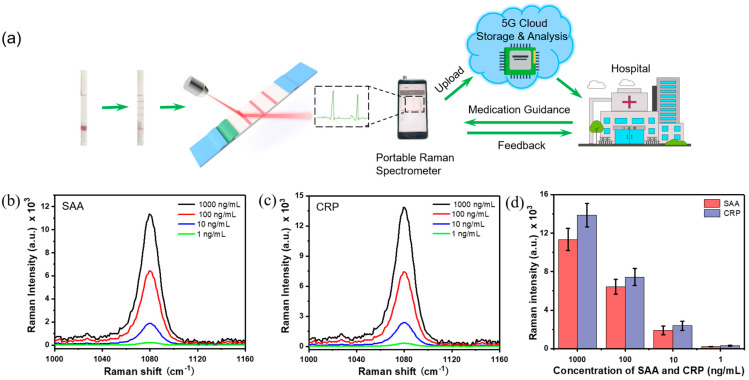
(**a**) Schematic illustration of telemedicine based on a portable Raman spectrometer after LFA. Averaged Raman Scheme 1079 cm^−1^ of two test lines for different concentrations of (**b**) SAA and (**c**) CRP measured by a portable Raman spectrometer. (**d**) The corresponding Raman intensities at 1079 cm^−1^ of two test lines.

We then used a portable Raman spectrometer to simultaneously and quantitatively detect CRP and SAA (Figure 5). We tested different concentrations (1, 10, 100, 1000 ng/mL) of SAA and CRP mixed solutions. Averaged Raman spectra of two test lines for different concentrations of SAA and CRP are shown in Figure 5b,c. The Raman intensities of the test lines gradually decreased with the concentration of SAA and CRP decreasing. As shown in Figure 5d, the minimum concentration of SAA and CRP when the Raman signal could be detected by a portable Raman spectrometer was 10 ng/mL. When the concentration of SAA and CRP was 1 ng/mL, the Raman intensities of SAA and CRP were both close to 0, indicating that the LODs of SAA and CRP were both 10 ng/mL when a portable Raman spectrometer was used to characterize the test lines. The LODs of SAA and CRP are lower than the previous LODs obtained when a confocal Raman spectrometer was used to characterize the test lines, which can be attributed to the sacrificing of sensitivity due to miniaturization of the portable Raman measurement devices. For instance, the maximum power of the portable Raman spectrometer used is only 500 mW, which is much lower than that of the confocal Raman spectrometer used before. Nevertheless, the results obtained by portable Raman spectrometer can satisfy practical use, since the LODs measured by using a portable Raman spectrometer are still lower than the LODs required in clinical practice for many diseases. For example, when the human body suffers from infectious diseases or sepsis, the concentration of SAA or CRP in the blood exceeds 10 ng/mL, and sometimes even exceeds 100 ng/mL or higher.

As shown in Figure 5a, we further envision a medical situation that can be easily realized in the near future to promote the application of the SERS-LFA strip-based POCT technology. After analytes such as the blood of patients with certain diseases are collected and quickly analyzed on-site by using our proposed SERS-LFA strips, the portable Raman spectrometer would acquire and upload the spectrum information to the cloud database. The cloud processor will store and analyze the spectrum information to obtain the corresponding processing results. Then, the processor sends the relevant processing results to the hospital in the meantime, and the doctor gives the patient corresponding medication guidance online as well based on the results provided by the cloud processor. The patient can also be tested again in the same way after drug treatment for long-term healthcare management without going to hospital for clinical testing. Therefore, using the portable cloud Raman spectrometer to characterize test strips is an effective and useful POCT tool for disease monitoring and diagnosis.

## 4. Conclusions

In summary, we prepared two core-shell Au^MBA^@mSiO_2_ SERS nanotags and proposed a SERS-LFA strip for the simultaneous and quantitative detection of SAA and CRP. We used a confocal Raman spectrometer and the brightness values analyzed by ImageJ software to quantitatively analyze the SERS-LFA strip. The LODs of using the Raman signal to quantitatively detect SAA and CRP were as low as 0.1 and 0.05 ng/mL, respectively, which was much lower than using the brightness value for quantification of the analytes. Regarding the POCT application, we utilized a smartphone-based portable Raman spectrometer to quantitatively analyze the SERS-LFA strips. The LODs of both SAA and CRP were 10 ng/mL, which is lower than the LOD required in clinical practice for most diseases, indicating their usefulness in practice. The current system can be a very promising POCT tool for disease monitoring and diagnosis. Through integration with a built-in 5G online healthcare management system, the proposed SERS-LFA method can be used as a diagnostic platform for the accurate analysis of a variety of biomarkers and provide medical guidance for the treatment of infection-related diseases that are widespread in remote areas lacking well-developed medical resources.

## Figures and Tables

**Table 1:** Comparison of our proposed SERS-LFA strip with other reported SAA/CRP detection methods.

Detection method	Analyte	LOD	Time	Reference
Protein microarrays	SAA	5.9 ng/mL	>2.5 h	Gul et al., 2007 [31]
SERS-LFA strip	CRP	0.01 ng/mL	20 min	Rong et al., 2018 [32]
Magnetoimmunosensor	CRP	8 ng/mL	20 min	Fernández et al., 2016 [33]
Fluorescent-LFA strip	CRP	0.5 ng/mL	15 min	Yang et al., 2020 [8]
SERS-LFA strip	SAA, CRP	0.1, 0.01 ng/mL	30 min	Liu et al., 2020 [6]
QD-based FLISA	SAA, CRP	2.39, 6.37 ng/mL	>1 h	Lv et al., 2019 [34]
SERS-LFA strip	SAA, CRP	0.1, 0.05 ng/mL	20 min	This work

## Data Availability

The data are available upon request from the corresponding authors.

## Supplementary Materials

The following are available online https://www.mdpi.com/article/10.3390/nano11061496/s1, Figure S1: TEM images of Au^MBA^@mSiO_2_ NPs with different TEOS usage (μL): (a) 140; (b) 160; (c) 180; (d) 200; (e) 400. Figure S2: (a) During the preparation of SERS nanotags, the zeta potentials of Au^MBA^@mSiO_2_, Au^MBA^@mSiO_2_-NH_2_, Au^MBA^@mSiO_2_-COOH NPs (N = 10) were measured. Relationship between the diameter of Au^MBA^@mSiO_2_ NPs and TEOS usage. (b) The relationship between the total Raman intensities of ten Au^MBA^@mSiO_2_ NPs and nanotags at 1079 cm^−1^ and the storage time of the nanoparticles. Figure S3a,b and c are the brightness curves of two test lines and one control line corresponding to the LFA strips in Figure 3a,d and Figure 4a, respectively. The area of the peak in the brightness curve is the brightness value. Figure S4a,b Averaged Raman spectra of two test lines for different concentrations of SAA and CRP corresponding to the SERS-LFA strips in Figure 4a measured by a confocal Raman spectrometer.
