# Supplementary material for: Simultaneous Detection of Inflammatory Biomarkers by SERS Nanotag-Based Lateral Flow Assay with Portable Cloud Raman Spectrometer
Source: Nanomaterials (Basel). 2021 Jun 5;11(6):1496. doi: 10.3390/nano11061496 (PMC8226521; doi:10.3390/nano11061496)
Supplement: Supplementary file 1 [file nanomaterials-11-01496-s001.zip › nanomaterials-1242965-supplementary.pdf]

Supplementary Materials

Simultaneous Detection of Inflammatory Biomarkers by SERS Nanotag-Based Lateral Flow Assay with Portable Cloud Raman Spectrometer

Yang Li [1], Xiaojia Liu [2,3], Jiuchuan Guo [4], Yueting Zhang [1], Jinhong Guo[4], Xinggui Wu[5,*], Bo Wang [1,*], and Xing Ma [2,3,*]

[1] School of Materials Engineering, Shanghai University of Engineering Science, Shanghai, 201620, China; liyang18124@163.com (Y.L.); 15039066601@163.com (Y.Z.)

[2] Sauvage Laboratory for Smart Materials, School of Materials Science and Engineering, Harbin Institute of Technology (Shenzhen), Guangdong, Shenzhen, 518055, China; 18b954089@stu.hit.edu.cn (X.L.)

[3] Shenzhen Bay Laboratory, No. 9 Duxue Road, Shenzhen 518055, China

[4] School of Communication and Information Engineering, University of Electronic Science and Technology of China, Chengdu 611731, China; uestc_gjc@163.com (J.G.)

[5] CloudMinds, Inc., Shenzhen Bay Science and Technology Ecological Park, Nanshan District, Shenzhen 100022, China; gelvin.wu@cloudminds.com (X.W.)

* Correspondence: gelvin.wu@cloudminds.com (X.W.); wangbo@sues.edu.cn (B.W.); maxing@hit.edu.cn (X.M.)

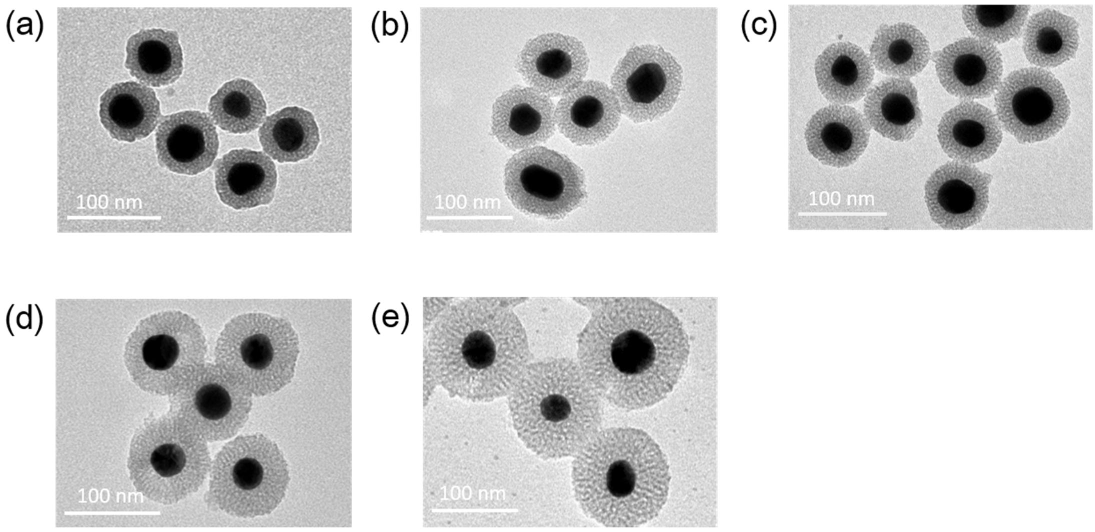

Figure S1: TEM images of $Au^{MBA}@mSiO_2$ NPs with different TEOS usage (μL): (**a**) 140; (**b**) 160; (**c**) 180; (**d**) 200; (**e**) 400.

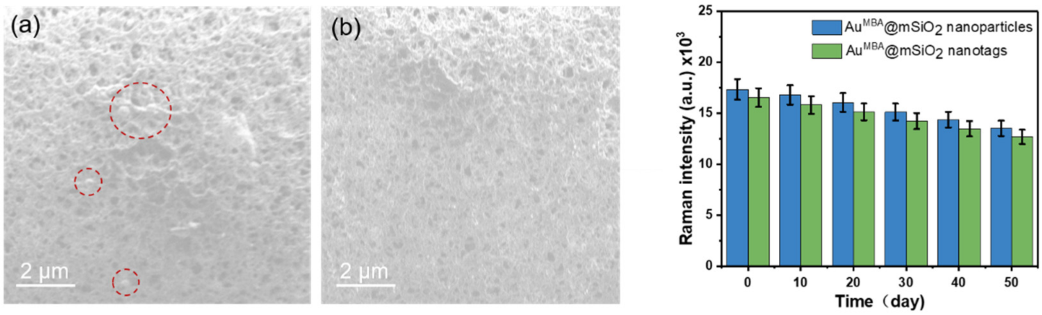

Figure S2: SEM images of LFA trips in Figure 4: (**a**) 1000 ng/mL, (**b**) 0 ng/mL. (**c**) The relationship between the total Raman intensities of ten Au^{MBA}@mSiO_2 nanoparticles and nanotags at 1079 cm^{-1} and the storage time of the nanoparticles.

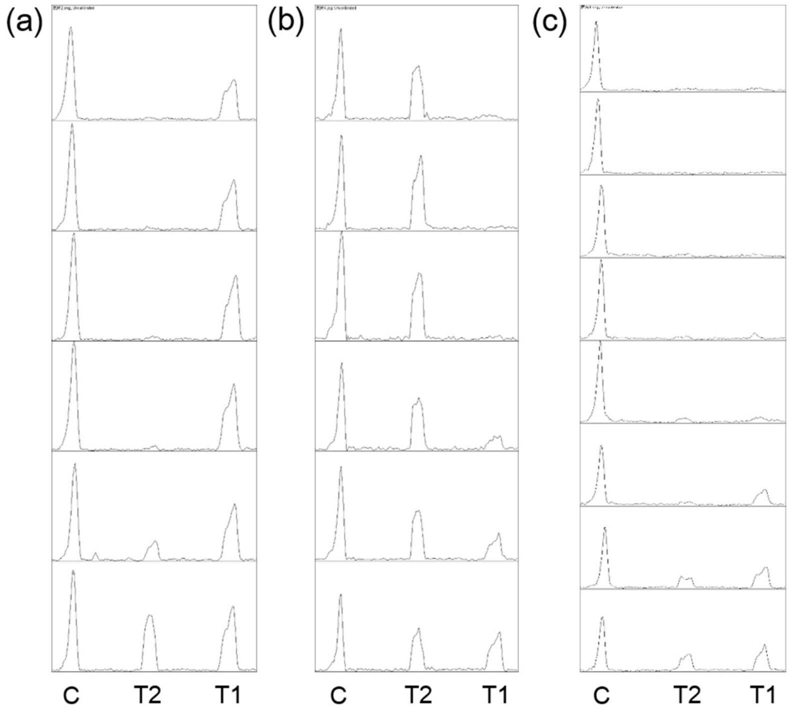

Figure S3: (**a**), (**b**), (**c**) are the brightness curves of two test lines and one control line corresponding to the LFA strips in Figure 3 (**a**), (**d**) and Figure 4 (**a**), respectively. The area of the peak in the brightness curve is the brightness value.

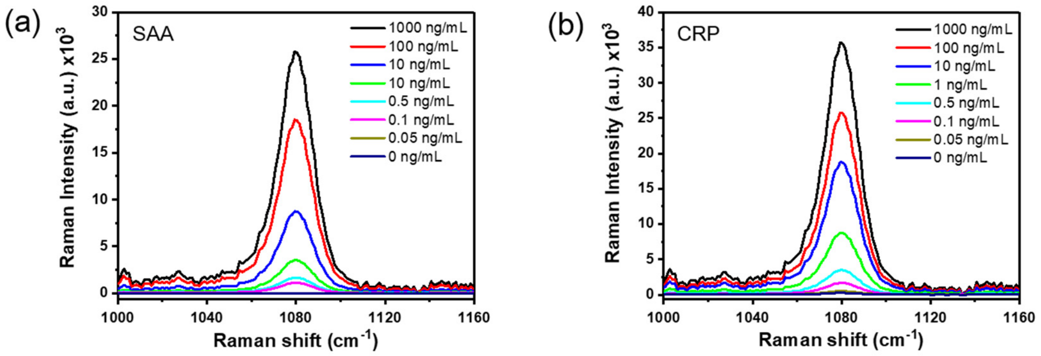

Figure S4：(**a**,**b**) Averaged Raman spectra of two test lines for different concentrations of SAA and CRP corresponding to the SERS-LFA strips in Figure 4 (**a**) measured by a confocal Raman spectrometer.
